# The impact of routine open nonsuction drainage on fluid accumulation after thyroid surgery: a prospective randomised clinical trial

**DOI:** 10.1186/1477-7819-10-72

**Published:** 2012-04-28

**Authors:** Peter M Neary, Owen J O’Connor, Azher Shafiq, Edel M Quinn, Justin J Kelly, Buckley Juliette, Ronan A Cahill, Josephine Barry, Henry P Redmond

**Affiliations:** 1 Department of Academic Surgery, Cork University Hospital, University College Cork, Cork, Ireland; 2 Department of Radiology, Cork University Hospital, University College Cork, Cork, Ireland

## Abstract

**Background:**

Thyroid drains following thyroid surgery are routinely used despite minimal supportive evidence. Our aim in this study is to determine the impact of routine open drainage of the thyroid bed postoperatively on ultrasound-determined fluid accumulation at 24 hours.

**Methods:**

We conducted a prospective randomised clinical trial on patients undergoing thyroid surgery. Patients were randomly assigned to a drain group (*n* = 49) or a no-drain group (*n* = 44) immediately prior to wound closure. Patients underwent a neck ultrasound on day 1 and day 2 postoperatively. After surgery, we evaluated visual analogue scale pain scores, postoperative analgesic requirements, self-reported scar satisfaction at 6 weeks and complications.

**Results:**

There was significantly less mean fluid accumulated in the drain group on both day 1, 16.4 versus 25.1 ml (*P*-value = 0.005), and day 2, 18.4 versus 25.7 ml (*P*-value = 0.026), following surgery. We found no significant differences between the groups with regard to length of stay, scar satisfaction, visual analogue scale pain score and analgesic requirements. There were four versus one wound infections in the drain versus no-drain groups. This finding was not statistically significant (*P* = 0.154). No life-threatening bleeds occurred in either group.

**Conclusions:**

Fluid accumulation after thyroid surgery was significantly lessened by drainage. However, this study did not show any clinical benefit associated with this finding in the nonemergent setting. Drains themselves showed a trend indicating that they may augment infection rates. The results of this study suggest that the frequency of acute life-threatening bleeds remains extremely low following abandoning drains. We advocate abandoning routine use of thyroid drains.

**Trial registration:**

ISRCTN94715414

## **Background**

Although no longer routinely utilised in colonic or biliary surgery [1,2] (because of an association with increased infection rates [3], discomfort [4] and hospital stay [5]), drain use following thyroid surgery remains common practice [6]. This may be because evidence negating their utility in terms of postoperative convalescence has been considered lacking in terms of both quality and quantity [5], especially with regard to nonsuction drains [5,7]. In addition, surgeons may continue to consider that a drain in the thyroid bed acts as an ‘early indicator’ of significant postoperative haemorrhage and provides a safeguard against compressive effects. Conversely, drains may augment scarring in a cosmetically sensitive area as well as infection risk and discomfort for no proven advantage, and they also add some time and expense to the end of the operation.

The continuing mismatch between common practice and the existing evidence base affirms the need for a further randomised clinical trial to examine whether drains provide any clinical benefit. We conducted a prospective randomised clinical trial to determine the impact of routine open drainage of the thyroid bed postoperatively on ultrasound-measured fluid accumulation. We hypothesised that if nonsuction drains have no significant effect on reducing fluid volumes in the thyroid bed following thyroid surgery, they are unlikely to have a beneficial effect if a life-threatening bleed occurs. Additional outcome measures assessed were visual analogue scale pain scores, postoperative analgesic requirements, self-reported scar satisfaction at 6 weeks and complications.

## **Methods**

### **Study design**

Following ethical approval, a prospective randomised clinical trial was conducted. The trial involved patients undergoing elective thyroidectomy (partial, subtotal, completion or total), with individuals being randomised at the end of the operation either to have a drain inserted or not. The primary study end point was volume of fluid accumulation in the thyroid bed assessed by ultrasound at 24 hours. Secondary end points assessed included postoperative pain (assessed on the basis of both visual analogue scale and analgesic requirement measurement) at 24 hours, fluid accumulation at 48 hours (ultrasound assessment) and length of hospital stay. Patients requiring sternotomy, neck dissection, younger than 18 years of age and a history of bleeding disorders were excluded. Consenting eligible patients were randomised by using a computer random number generator and, to do this, were consecutively allocated a numbered envelope indicating to which group they were assigned (either a drain or no-drain group). Both surgeon and patient were blinded from group allocation until immediately prior to wound closure.

### **Operating protocol**

All procedures were carried out under the direct supervision of the same senior surgeon. Complete haemostasis was ensured throughout the surgery by using a combination of meticulous technique, point diathermy and suture ligation. Only when the operation was entirely completed and just at the point of wound closure was the randomisation envelope opened. In the drain group, a Penrose drain (Irish Hospital Supplies Ltd, Bray, Co Wicklow, Ireland) was brought out through a separate wound. A nonsuction open drain was chosen because previous reports have described closed suction drainage as being futile in this instance because of the propensity to block [8]. Intraoperative analgesia consisted of intravenous fentanyl, morphine, paracetamol and diclofenac if not contraindicated.

### **Postoperative assessments**

Type and length of surgery, size [9] and weight of specimen, indication for surgery and histological diagnosis were documented.

#### ***Ultrasound assessment of fluid***

Neck ultrasound using a B mode with linear frequency of 17 MHz (Philips Healthcare, Eindhoven, the Netherlands) was performed at 24 and 48 hours following the surgery by one single operating radiologist. Maximal three-dimensional diameters of fluid collection in the thyroid bed were measured in triplicate, and the average was multiplied by 0.52 to calculate fluid volume [9].

#### ***Postoperative pain assessment***

Postoperative pain during the hospital stay was managed primarily with paracetamol, and diclofenac and morphine were reserved for breakthrough analgesia. A record was kept of analgesic dosing requirements on each post-operative day. Visual analogue scale pain scoring was also used to gauge patient discomfort at 24 hours postoperatively.

Postoperative complications, including infection, acute life-threatening postthyroidectomy bleed, neck haematoma (whether requiring intervention or not) and symptoms of hypocalcaemia, were recorded both throughout the hospital stay and at the first scheduled clinic appointment after surgery (6 weeks postoperatively). A wound infection was diagnosed if purulent discharge exuded from the wound [10] or a painful, spreading erythema indicative of cellulitis existed. At six weeks, patient satisfaction with scarring was assessed by subjective patient ranking on a scale from 0 to 10.

### **Power calculation**

The power calculation was based on the hypothesis that mean fluid accumulation in the thyroid bed at 24 hours would increase from an expected 35 ± 17 ml [11] to 45 ml or greater in the drain versus no-drain groups, respectively. We estimated that allocating a minimum of 44 patients to each group would give at least 80% power to detect a difference in fluid volume in both groups, using an independent samples *t*-test with a two-sided type I error rate of 0.05 (nQuery Advisor v4 software; Statistical Solutions, Cork, Ireland). We hypothesised that if nonsuction drains have no significant effect on reducing fluid volumes in the thyroid bed following thyroid surgery, they would be unlikely to have a beneficial effect if a life-threatening bleed were to occur.

### **Statistical analysis of results**

The data were analysed using SPSS version 16 software (SPSS, Inc, Chicago, IL, USA). Fisher’s exact test was used to determine whether there were significant differences between the groups of interest. Tests of normality were conducted using the Kolmogorov-Smirnov test. Mean values were compared for statistical significance using Student’s *t*-test. A value of *P* < 0.05 was taken as the level that ascribed statistical significance.

## **Results**

**Figure 1:**
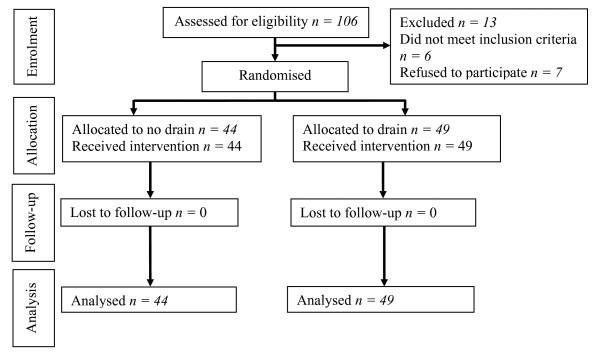
CONSORT flow diagram for the trial.

Ninety-three patients were recruited for the study. Five other patients had either a sternotomy or neck dissection, one was younger than 18 years of age and seven individuals declined to participate (see Figure 1 for the CONSORT flow diagram). There were 49 patients assigned to the drain and 44 assigned to the no-drain group. The mean ages for these groups were 53.0 years (±15.1) and 50.5 years (±14.4), respectively, and the male-to-female proportions were 0.17 and 0.13, respectively. Further patient data, including length of surgery, histological weight, histological size, use of anticoagulant or antithrombotic therapy, indication for surgery and histological diagnosis, showed no significant intergroup differences (see Table 1).

**Table 1:** Summary of characteristics of surgery and histology in drain versus no-drain group

**Measurement parameter**	**Drain group**	**No drain group**	***P*-value**
Type of surgery (*n*)			0.497
Lobectomy and isthmusectomy	30	30	
Total	13	9	
Completion	6	5	
Mean length of surgery (minutes)	106.5	107.3	0.875
Mean histological weight (g)	63.3	54.4	0.537
Mean histological size (cm^3^)	71.3	77.0	0.833
Patients taking anticoagulants (*n*)	6	5	0.865
Indication for surgery (*n*)			
Suspicious nodule for cancer	44	42	0.215
Hypervascular disorders			
Graves disease	2	1	0.541
Hashimoto’s thyroiditis	0	0	-
Hyperthyroidism	3	1	0.289
Histological diagnosis (*n*)			
Cancer	14	7	0.062
Hypervascular disorders			
Graves disease	1	0	-
Hashimoto’s thyroiditis	6	3	0.275

No significant intergroup differences were observed (two-paired Student’s *t*-test and Fisher’s exact test).

A tabulated summary of the study end points is shown in Table 2, which shows a comparison of the drain versus no-drain groups. We found no significant differences between the groups with regard to length of stay, visual analogue Scale pain score and analgesic requirements. At both measured time points, fluid accumulation in the drain group was significantly less than in the no-drain group (16.4 ± 9.3 ml versus 25.1 ± 17.7 ml, *P* = 0.005; and 18.4 ± 9.9 ml versus 25.7 ± 18.6 ml, *P* = 0.026, at 24 and 48 hours, respectively). There were four wound infections in the drain group and one wound infection in the no-drain group. This finding was not statistically significant (*P* = 0.154). The one patient in the no-drain group who developed a wound infection had required ultrasound-guided drainage of an unresolved haematoma 2 weeks following that surgery. Of the five infections diagnosed, all resolved following a course of antibiotics (one patient in each group required hospital admission for intravenous antibiotics).

**Table 2:** Summary of end points analysed in drain versus no-drain groups

**Measurement parameter**	**Drain group**	**No drain group**	***P*-value**
Mean length of postoperative stay (days)	2.3	2.1	0.477
Mean pain score (maximum = 10)	2.9	3.0	0.803
Median postoperative analgesic requirements as per World Health Organisation pain ladder	Level II	Level II	0.747
Mean fluid accumulation on ultrasound (ml)			
24 hours	16.4	25.1	0.005
48 hours	18.6	25.7	0.026
Complications (*n*)			
Symptoms hypocalcaemia	4	5	0.444
Shortness of breath	0	0	-
Wound infection	4	1	0.154
Postthyroidectomy bleed	0	0	-
Haematoma requiring drainage	0	1	-
Other (RTI)	0	1	-
Mean satisfaction with scar (maximum = 10)	8.0	8.2	0.540
Satisfaction with overall hospital stay (maximum = 10)	8.6	8.6	0.992

Significantly more fluid accumulation was observed in the no-drain group at both 24 and 48 hours (two-paired Student’s *t*-test and Fisher’s exact test).

Table 3 summarises fluid accumulation observed on ultrasounds in the drain and no-drain groups at 24 hours according to type of surgery, size of gland and histological diagnosis. There was no significant difference in fluid accumulation between these subgroups at 24 hours in the drain group. However, the no-drain group showed a significantly increased fluid accumulation following total thyroidectomy (*P* = 0.021) and significantly less following completion thyroidectomy (*P* = 0.026) compared with the other types of surgery performed. The no-drain group also showed significantly more fluid accumulation in resected glands above 36.5 cm (*P* = 0.012) [3] (the median size of glands in this study). Neither a malignant diagnosis nor the presence of a hypervascular disorder (Graves disease or Hashimoto’s thyroiditis) imparted any significant differences in fluid accumulation in either group. Furthermore, patients taking antithrombotic agents (exclusively aspirin in the study cohort) prior to surgery showed no significant increases in fluid accumulation postoperatively (although all had discontinued their medication as per routine advice 1 week prior to surgery). RTI signifies respiratory tract infection.

**Table 3:** Subgroup analysis of fluid accumulation on the basis of ultrasonography performed at 24 hours in the drain and no-drain groups with respect to type of surgery, size of gland and histological diagnosis

	**Mean fluid accumulation at 24 hours (ml)**			
**Measurement parameter**	**Drain group**	***P*-value**	**No-drain group**	***P*-value**
Type of surgery				
Lobectomy and isthmusectomy	16.3	0.969	22.4	0.309
Total	18.7	0.450	42.7	0.021
Completion	12.2	0.271	8.9	0.026
Histological size				
Below overall median size (36.5 cm^3^)	16.2		19.5	
Above overall median size (36.5 cm^3^)	20.0	0.166	36.7	0.012
Histological diagnosis				
Cancer	17.9	0.560	20.2	0.483
Hypervascular disorders	14.7	0.450	15.1	0.310
Other (benign) diagnoses	15.9	0.751	18.0	0.900

*P*-values (two-paired Student’s *t*-test) were derived by comparing the ultrasound-based volume at the corresponding end point to the ultrasound-based volume of the remaining patients within the same group (that is, drain group versus no-drain group)

## **Discussion**

The utility of thyroid drain insertion has recently come under scrutiny. Reviews on this topic by Samraj *et al*. [5] and Kennedy *et al*. [7] have highlighted that no high-quality study has shown any clinical benefit for its use. Thus far, however, the evidence has not been sufficient to dissuade all surgeons to definitively advocate abandoning thyroid drains.

Though a study examining the effect of drains on the frequency and severity of life-threatening bleeds following thyroidectomy would be ideal, life-threatening bleeds are extremely rare. Hence such a study would unfortunately take numerous years and a vast amount of patients to accumulate sufficient data to complete and is outside the remit of our present study. Instead, we evaluated whether nonsuction thyroid drains are effective in reducing fluid accumulation in the thyroid bed following surgery. We hypothesised that if nonsuction drains have no significant effect on reducing fluid volumes in the thyroid bed following thyroid surgery, they are unlikely to have a beneficial effect if a life-threatening bleed occurs. This study shows that nonsuction drain insertion significantly reduces fluid accumulation in the thyroid bed postoperatively. This finding dispels previous concerns [12] that most thyroid drains are redundant on the basis that they frequently block. Our findings contrast with the findings of Khanna *et al*. [8], who found no significant difference in fluid accumulation measured by ultrasound in a closed-suction drain group compared to a no-drain group. Hence open drains appear to be more successful than closed suction drains in draining the thyroid bed.

Although nonsuction drains successfully reduced fluid accumulation in the thyroid bed following surgery, interestingly, this did not confer any obvious clinical benefit to the patients in this study. However, this study shows that if a life-threatening bleed did occur, an open nonsuction drain, in contrast to a suction drain, would most likely drain fluid, which might result in a considerable benefit regarding patient outcome. We did not address the issue of the advantage that drains may actually confer in the setting of an acute life-threatening haemorrhage because of the absence of its occurrence in the patient cohort. It is reassuring, however, that no life-threatening bleed occurred in the no-drain group, thus reaffirming that this complication remains low. The observation that infection rates were four times higher in the drain versus the no-drain group is further supportive of foregoing routine drain insertion. Although the study is underpowered to ascribe statistical significance to this observation, investigators have observed similar findings in previous studies [3].

## Conclusion

In conclusion, fluid accumulation after thyroid surgery is significantly lessened by nonsuction drains. However, the results of this study suggest that the frequency of acute life-threatening bleeds remains low following abandoning drains. This study, in combination with other data, shows that routine use of thyroid drains can safely be abandoned.

## **Competing interests**

The authors have no conflict of interest with regard to the materials, methods or data reported in this paper.

## Authors’ contribution

All authors contributed to hypothesis and research design, data collection and manuscript editing.
